# Computational Drug Target Screening through Protein Interaction Profiles

**DOI:** 10.1038/srep36969

**Published:** 2016-11-15

**Authors:** Santiago Vilar, Elías Quezada, Eugenio Uriarte, Stefano Costanzi, Fernanda Borges, Dolores Viña, George Hripcsak

**Affiliations:** 1 Department of Biomedical Informatics, Columbia University Medical Center, New York, NY 10032, USA; 2 Department of Organic Chemistry, Faculty of Pharmacy, University of Santiago de Compostela, 15782, Santiago de Compostela, Spain; 3 CIQUP, Department of Chemistry & Biochemistry, Faculty of Sciences, University of Porto, 4169-007, Porto, Portugal; 4 Department of Chemistry, American University, 20016 Washington, DC, USA; 5 Department of Pharmacology, CIMUS, University of Santiago de Compostela, 15782, Santiago de Compostela, Spain

## Abstract

The development of computational methods to discover novel drug-target interactions on a large scale is of great interest. We propose a new method for virtual screening based on protein interaction profile similarity to discover new targets for molecules, including existing drugs. We calculated Target Interaction Profile Fingerprints (TIPFs) based on ChEMBL database to evaluate drug similarity and generated new putative compound-target candidates from the non-intersecting targets in each pair of compounds. A set of drugs was further studied in monoamine oxidase B (MAO-B) and cyclooxygenase-1 (COX-1) enzyme through molecular docking and experimental assays. The drug ethoxzolamide and the natural compound piperlongumine, present in Piper longum L, showed hMAO-B activity with IC_50_ values of 25 and 65 μM respectively. Five candidates, including lapatinib, SB-202190, RO-316233, GW786460X and indirubin-3′-monoxime were tested against human COX-1. Compounds SB-202190 and RO-316233 showed a IC_50_ in hCOX-1 of 24 and 25 *μ*M respectively (similar range as potent inhibitors such as diclofenac and indomethacin in the same experimental conditions). Lapatinib and indirubin-3′-monoxime showed moderate hCOX-1 activity (19.5% and 28% of enzyme inhibition at 25 μM respectively). Our modeling constitutes a multi-target predictor for large scale virtual screening with potential in lead discovery, repositioning and drug safety.

Discovery of new targets for molecules is of great interest in drug design and development^1^,^2^. High throughput screening and computational methods for virtual screening have been successfully used to search for novel targets in extensive libraries of compounds^3^,^4^. Moreover, when compounds of interest are drugs in the market, the detection of new targets have implications in drug repositioning and safety. Drugs already in the market or experimental drugs can also be excellent lead compounds for optimization of the activity in the further development process. In recent years different computational methods have been published to discover and characterize new drug-target interactions^5^,^6^. In the big data era, these computational approaches can acquire great potential in drug discovery due to the vast amount of information we can manage currently. Molecular similarity has been a strategy widely applied to discover new molecules that bind a specific target^7^,^8^,^9^. Molecular similarity can be assessed using different types of approaches. 2D molecular structure similarity can be calculated through methods such as molecular fingerprints. As an example, Keiser *et al.* related proteins based on 2D chemical structure similarity of their ligands^10^. Their approach, denominated SEA (Similarity Ensemble Approach), offered great results in drug-target discovery and yielded sets of drug-target associations confirmed experimentally^10^,^11^. A more complex similarity approach takes into account 3D molecular structure information. Our research group has developed large scale predictive modeling through the implementation of 3D drug structure similarity into biological knowledge data sources^12^,^13^. However, alternative measures to the molecular structure can be calculated to evaluate the similarity between drugs. Target profile, drug-drug interaction and adverse effect profiles represent drug biological fingerprints that can be compared^14^. In fact, comparison of drug similarity using side effect profiles yielded methods with great applications in identifying novel drug-target associations^15^,^16^. Common molecular pathology has also been exploited in drug discovery under the idea that two diseases or indications could share the same molecular mechanisms modulated by the drugs´ action^17^,^18^,^19^. Disease similarity based on shared drug therapies was already implemented to generate models to discover new drug-indication associations^20^. Integration of heterogeneous biological data, such as drug similarity profiles with protein similarity, also yielded good performance in drug-target prediction^16^,^21^. Other bioinformatics approaches showed the potential of comparing gene expression profiles in microarrays data to discover new associations between drugs, targets, pathways perturbations and diseases^22^,^23^,^24^.

In this article, we developed a new approach for target based-virtual screening comparing a large data of molecules, including drugs already on the market, experimental drugs and natural compounds, based on their target interaction profiles. The predictor described here is a large scale predictor for multiple target screening developed with extensive protein binding data extracted from ChEMBL (including 449,996 compound-target cases). A set of candidates including drugs and natural compounds were selected to further study through molecular docking and experimental validation in the human monoamine oxidase B (hMAO-B) enzyme and the human cyclooxygenase-1 (hCOX-1). The flowchart of the main steps carried out in this study is shown in Fig. 1.

## Results

### Modeling target interaction profiles for drug virtual screening

**Figure 2:**
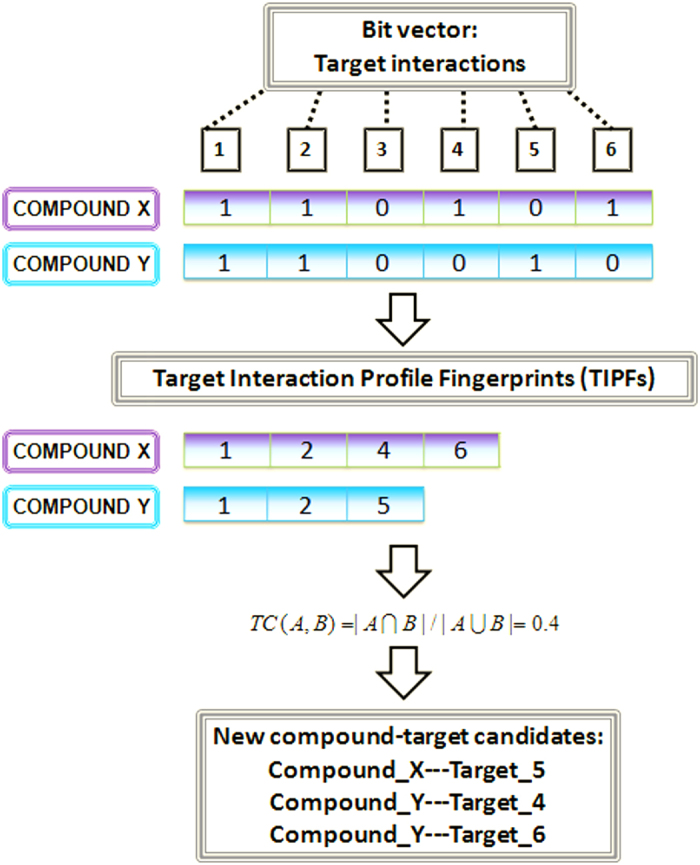
Representation of Target Interaction Profile Fingerprints (TIPFs), computation of the similarity through the Tanimoto coefficient (TC), and generation of new putative target interaction candidates.

We developed a model for multiple target virtual screening to discover novel targets for drugs. For this purpose we calculated Target Interaction Profile Fingerprints (TIPFs) for the compounds in ChEMBL data source^25^. Tanimoto coefficient (TC) between all the pairs of compounds was calculated based on the target interaction profiles (see Fig. 2).

**Figure 3:**
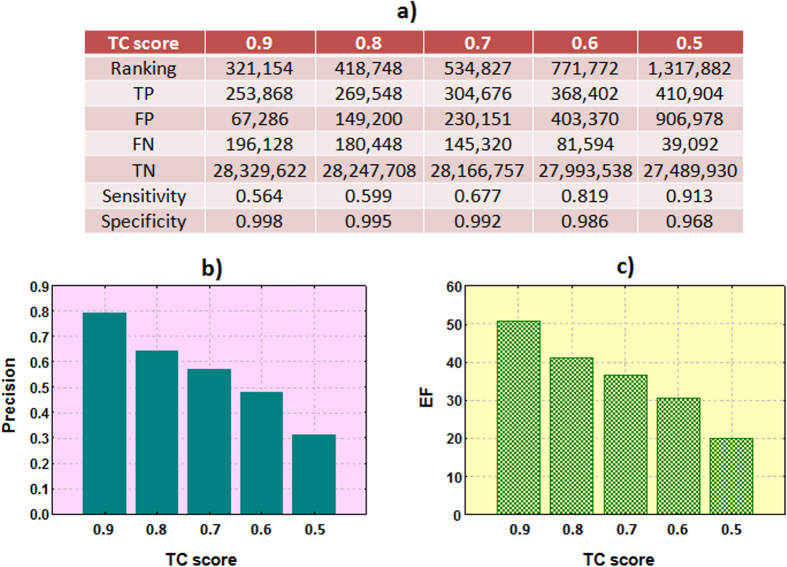
Sensitivity/specificity (**a**), precision (**b**) and EF (**c**) at different thresholds of the TC (TP: true positives, FP: false positives, FN: false negatives, TN: true negatives, EF: enrichment factor).

The predictor associated the TC score with the compound-target candidates exchanging targets in each pair of compounds. When the same compound-target association is generated from the comparison of different pairs, only the maximum score is retained. In that manner, each possible compound-target candidate is associated with the maximum similarity score calculated against the compounds associated in ChEMBL with the same target. The predictor yielded compound-target associations already in the initial ChEMBL data (positive controls) but also new putative compound-target associations interesting to further study. Due to the big amount of data (28,846,904 possible compound-target combinations) and to simplify the process we retained only the compound-target associations with TC ≥ 0.5. Sensitivity, specificity, precision and enrichment factor (EF) at different thresholds were reported (see Fig. 3). Results showed high degree of recovery of the active compounds.

**Figure 4:**
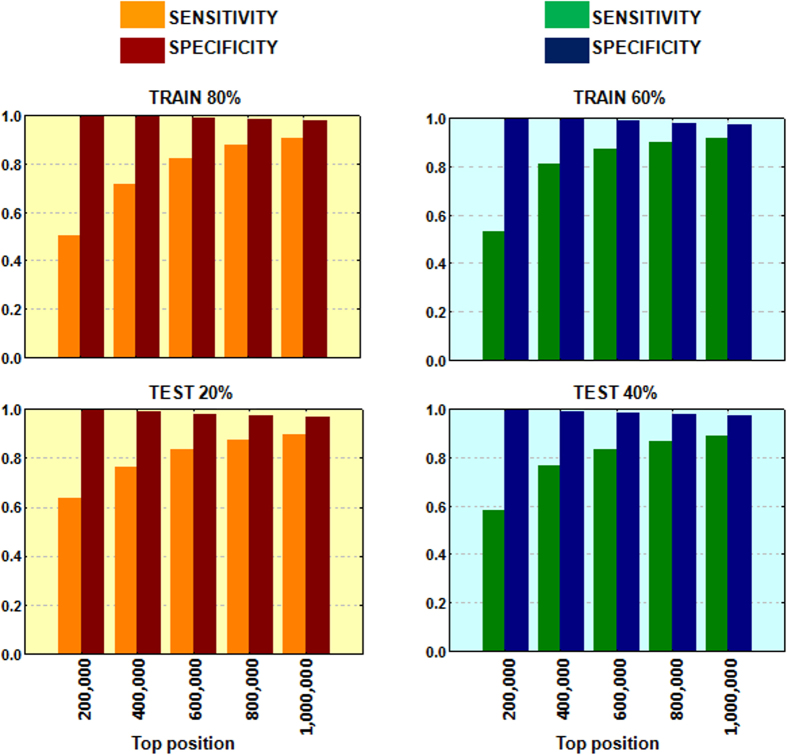
Sensitivity and specificity values in the hold-out validation series at different thresholds (from top 200,000 to top 1,000,000).

Additional evaluation was performed through hold-out validation series. With this purpose, and before fingerprint calculation, we divided the initial data in two series that were called training and testing subsets. We first included 80% of the initial data into a training set and the 20% in a test. Second, we introduced only the 60% of the data in the training whereas the 40% was included in the test set. Selection of the data was carried out randomly. Results are shown in Fig. 4. The stability of the multi-target predictor was not affected by the division of the data showing the modeling robustness. However, further evaluation of the system was necessary to prove the predictive power. For this reason, a set of candidates was selected for further validation.

Out of the total number of compound-target candidates with TC ≥ 0.5 we selected drugs already on the market, experimental drugs and natural products. The final set of drug-target candidates comprised 267,314 associations with a TC ≥ 0.50. Out of the total number, 94,253 associations were already present in the reference standard, 1,192 were inactive in ChEMBL and 171,869 are new associations detected by the modeling. To validate our results, the candidates generated for hMAO-B and hCOX-1 were selected to carry out molecular docking simulations and to evaluate their *in vitro* hMAO-B and hCOX-1 inhibitory activities. Thirteen drugs on the market, experimental drugs or natural compounds were pointed out by the model as hMAO-B candidates. In the case of hCOX-1, the model pointed out 384 candidates. A second more restrictive selection of the candidates for both systems was carried out through molecular docking studies.

### Molecular docking simulations

Molecular docking calculations were performed in the human MAO-B and COX-1 (organism *Ovis aries*, since not human protein was available) to select a subset of candidates for experimental evaluation and to study the key interactions responsible for the binding between drugs/natural compounds and the proteins. Docking protocol in hMAO-B was previously established by our research group^26^,^27^,^28^. Since some of the candidates are generated due to their similarity against the antiepileptic drug zonisamide, the crystal structure of the hMAO-B in complex with zonisamide (PDB: 3PO7)^29^ was used to carry out our simulations. Five water molecules were retained in the cavity of the protein (see Methods). We carried out docking calculations using Glide SP (standard precision) from Schrödinger^30^ (see docking validation in Methods). Additional docking calculations with/without water molecules in the 3PO7 pocket as well as using an alternative crystallized hMAO-B structure (PDB: 2V60)^31^ were performed and corroborated the obtained results.

**Figure 5:**
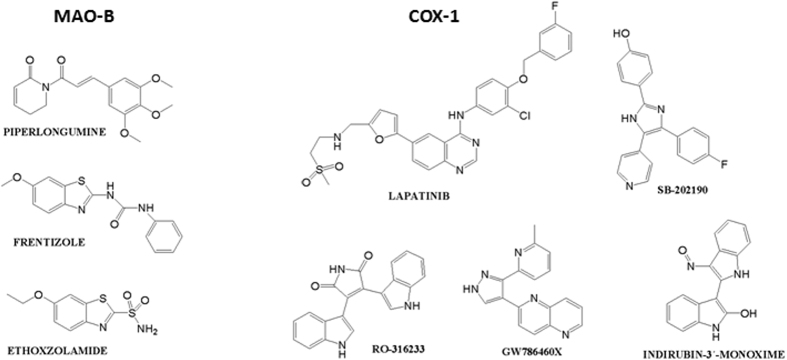
Structure of compounds selected for hMAO-B and hCOX-1 experimental validation.

**Figure 6:**
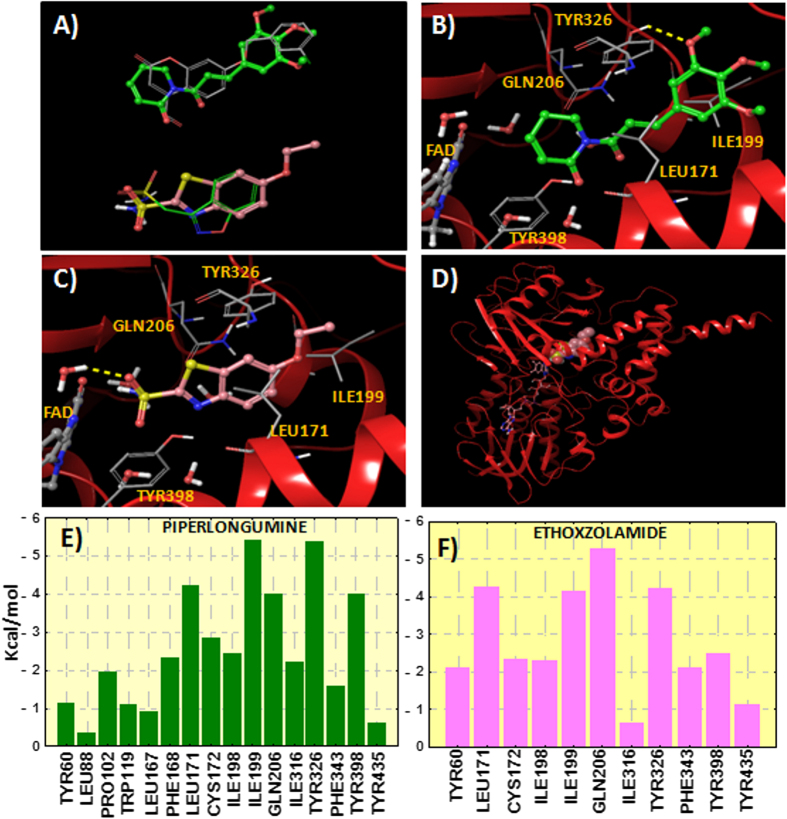
(**A**) Comparison between the theoretical poses determined for piperlongumine (green carbons) and ethoxzolamide (pink carbons) and the co-crystallized inhibitors c17 (2V60) and zonisamide (3PO7) inside the hMAO-B. (**B**) Binding mode for piperlongumine extracted from the hMAO-B docking. Hydrogen bond is represented in yellow color. Protein ribbons are partially omitted for clarity. (**C**) Binding mode determined for ethoxzolamide inside the hMAO-B. (**D**) Perspective of the whole hMAO-B enzyme with ethoxzolamide docked to the protein (FAD cofactor in stick representation and ethoxzolamide in CPK). (**E**) Residue interaction energy scores between hMAO-B and piperlongumine. (**F**) Residue interactions with ethoxzolamide. Contribution of the residues in a distance of 4 Å from the ligands is plotted. Interaction energy is calculated as the sum of the contributions of Coulomb, *van der Waals* and hydrogen bonding.

The 13 candidates selected previously through our TIPF virtual screening were docked to the hMAO-B. Three top candidates according to the different docking scoring calculations were selected for hMAO-B binding assays (see Table 1 and Fig. 5 with the candidates selected for experimental evaluation and the most similar compound according to our TIPF model). The score achieved by the molecular docking is shown in Table S1 of the Supporting Information. The best ranked candidate was piperlongumine, also called piplartine, a natural compound present in long pepper, (Piper longum L.). Piperlongumine orientated the dihydropyridinone ring towards the FAD cofactor whereas the trimethoxyphenyl group was directed towards the hydrophobic entrance cavity (see Fig. 6). The natural compound established a hydrogen bond by anchoring the methoxy group with the residue Tyr326. However, the methoxy group should have to displace a crystallized water molecule in the hMAO-B pocket and this fact could limit the ligand binding. Two more candidates also showed potential from the point of view of the molecular docking simulations. Frentizole is a benzimidazoleurea drug with immunosuppressive properties and ethoxzolamide is a drug, currently discontinued by the FDA^32^, used as diuretic and for glaucoma treatment. Ethoxzolamide was detected to be similar to zonisamide according to our TIPF model. The binding mode extracted from ethoxzolamide docking also presented some similarity with the co-crystallized zonisamide inside the hMAO-B (PDB: 3PO7)^29^. The sulfonamide group was oriented towards the FAD in a similar position as zonisamide (see Fig. 6) and established a hydrogen bond with the co-crystallized water molecule HOH545.

Additionally, we calculated the contribution of the different residues of the hMAO-B to the interaction energy with piperlongumine and ethoxzolamide (see Fig. 6). The residue contribution was calculated as the sum of three energetic terms: Coulomb, *van der Waals* and hydrogen bond energies. Both compounds were found to interact preferentially with residues Leu171, Ile199, Gln206, Tyr326 and Tyr398. The important role of the cited residues in ligand binding, such as Ile199 and Tyr326, was previously reported through molecular docking and site-directed mutagenesis studies^27^,^33^.

**Figure 7:**
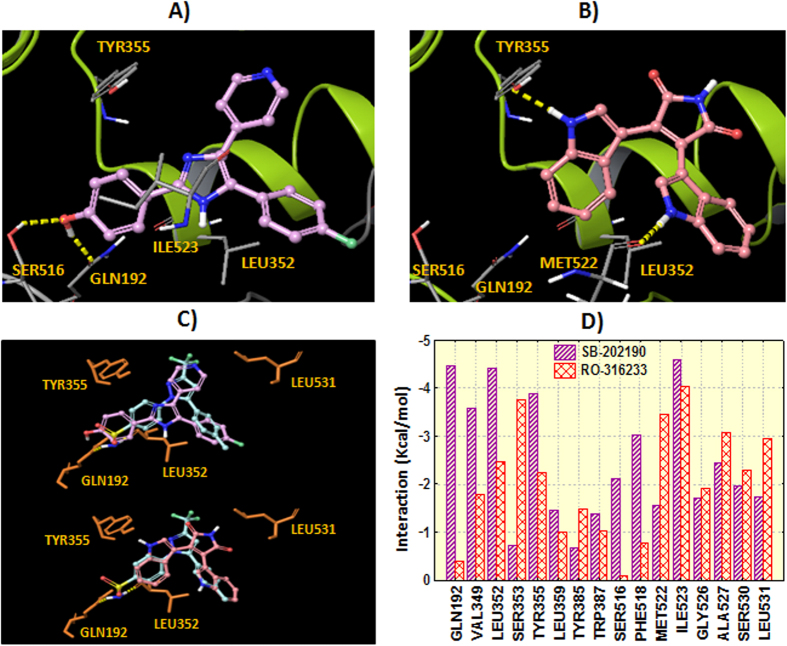
Hypothetical binding mode extracted from docking in the COX-1 for SB-202190 (panel A) and RO-316233 (panel B). Hydrogen bonds are represented in yellow dashes. Protein ribbons are partially omitted for clarity. Panel C shows the comparison between the theoretical docking poses determined for the ligands (SB-202190: plum carbons, RO-316233: pink carbons) and the co-crystallized celecoxib (turquoise carbons). Residue interactions with the ligands (sum of Coulomb, *van der Waals* and hydrogen bonding) are shown in panel D.

Molecular docking in the COX-1 (*Ovis aries*) was performed through Glide SP^30^ using the crystal structure 3KK6 (PDB code)^34^. Validation of the docking protocol is explained in the Methods section. Five experimental/approved drugs were selected for further biological evaluation taking into account docking ranking and commercial availability (see Fig. 5 with the molecular structures and Table S2 with the docking scoring, ranking and the most similar compound in our ChEMBL reference standard). The candidates selected for experimental validation are: lapatinib, a drug approved by the FDA for breast cancer patients, SB-202190, an inhibitor of p38 MAP kinase, RO-316233, a core structure of different biologically active molecules, GW786460X, an ATP-competitive inhibitor of TGF-β type I receptor, and indirubin-3′-monoxime, a potent inhibitor of glycogen synthase kinase 3β and cyclin-dependent kinases. Molecular docking showed that lapatinib and SB-202190 established hydrogen bonds with the residues Ser516 and Gln192. Similar interactions are described for celecoxib in the crystal structure 3KK6^34^ where the sulfonamide group interacts with residues Leu352 and Gln192. Indole groups in compound RO-316233 play an important role by anchoring to residues Tyr355 and Met522 through hydrogen bonding. Figure 7 shows the hypothetical binding modes extracted from molecular docking for the candidates SB-202190 and RO-316233. Additionally, Fig. 7 shows the superposition between the co-crystallized celecoxib and the binding modes extracted from docking along with residue interactions with the candidates SB-202190 and RO-316233.

### Experimental data validation

We evaluated three candidates, piperlongumine, frentizole and ethoxzolamide, for their ability to inhibit the isoform B of the human monoamine oxidase (hMAO-B). Inhibition of the isoform hMAO-A was also tested to prove that the candidates pointed out by the model are selective against the hMAO-B (the 3 candidates were not pointed out by our model as inhibitors of the A isoform). On the other hand, we evaluated five candidates against hCOX-1 (lapatinib, SB-202190, RO-316233, GW786460X and indirubin-3′-monoxime). To assess the ability of our system to detect novel lead compounds we cross-tested our candidates. The three candidates selected for hMAO-B were evaluated against hCOX-1 whereas the five hCOX-1 candidates were tested against hMAO-B (control tests). The corresponding IC_50_ values are shown in Tables 2 and 3.

Two out of our three drug candidates displayed inhibitory activity towards hMAO-B. Moreover, they were hMAO-B selective since none of them showed activity in the A isoform. Ethoxzolamide was found to be the most potent hMAO-B compound with an IC_50_ of 25 *μ*M that is in a similar range as zonisamide (*K*_i_ = 3.1 *μ*M and IC_50_∼25 *μ*M)^35^,^36^, the reference compound in our TIPF model. Although zonisamide exhibits a moderate hMAO-B potency, it has been described to improve the clinical outcome in Parkinson’s disease (PD) patients, namely when used as coadjuvant with other PD drugs^35^,^37^. Due to its activity in neuroprotection, zonisamide is in clinical Phase III for its use in PD^38^. Our drug, ethoxzolamide, showed similar moderate activity in hMAO-B, although further studies are necessary to confirm the human neuroprotective potential. However, ethoxzolamide could be a good candidate also as a lead compound for optimization series. Piperlongumine showed moderate hMAO-B activity with a IC_50_ of 65 *μ*M. Further studies are needed to evaluate if the natural compound piperlongumine shows neuroprotective effects in humans. The natural compound present in long pepper, (Piper longum L.) can be considered an interesting lead for further optimization steps. Frentizole, in our experimental conditions, did not display a noticeable hMAO-B activity at the higher tested concentration (100 μM).

Two of the five compounds selected for hCOX-1 evaluation showed good inhibitory activity values (see Table 3). The most active compounds in the hCOX-1 evaluation were SB-202190 and RO-316233 with a IC_50_ of 24 and 25 *μ*M, respectively. It is worth noting that potent, well-known hCOX-1 inhibitors in the range of nM in some COX assays, such as diclofenac and indomethacin, showed similar IC_50_ values under the same experimental conditions (IC_50_ of 18 and 12 *μ*M respectively)^39^. Compounds such as lapatinib and indirubin-3′-monoxime also showed moderate hCOX-1 activity although IC_50_ values were not obtained due to compounds solubility limitations in the experimental assay (see Table 3 with % of enzyme inhibition). GW786460X is inactive against hCOX-1 at 100 μM (highest concentration tested).

When we cross-tested our candidates (3 hMAO-B candidates were evaluated against hCOX-1 whereas 5 hCOX-1 candidates were tested against hMAO-B), none of the compounds presented activity in these experimental assays (inactive at 100 μM, highest concentration tested). We compared both proportions from a statistical point of view and a significant difference was observed (*p*-value = 0.006, Fisher’s exact test). In our rationally designed assays, four out of six compounds were moderately active (two compounds were not taken into account due to the impossibility of calculating IC_50_ although they presented moderate activity). However, none of the control evaluations were satisfactory from the activity point of view (0 out of 11 including hMAO-A, hMAO-B and hCOX-1). The data supports the predictive ability of our method. Our method was able to detect novel structures that can be considered interesting leads for further activity optimization.

## Discussion

**Figure 8:**
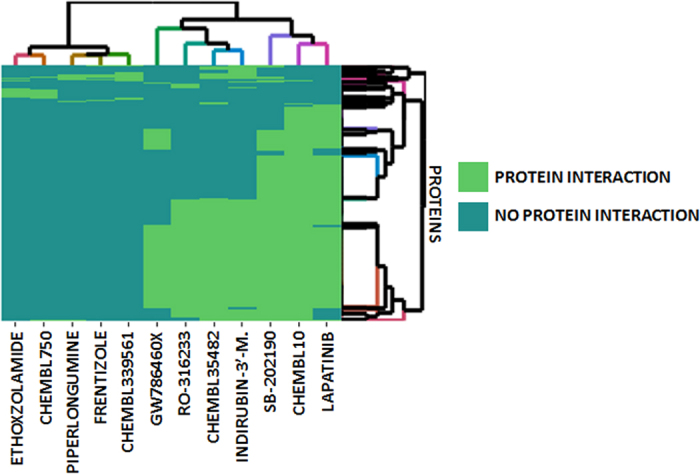
Heatmap of target association profiles for the hMAO-B and hCOX-1 candidates along with their reference compounds in ChEMBL. Targets described for both types of candidates are included.

In our modeling each pair of compounds was compared based on their protein interaction profiles extracted from ChEMBL database (see Fig. 8 with a heatmap of target association profiles for the evaluated candidates). The modeling assigned a score to each compound-target combination based on the maximum similarity against the set of compounds that bind the target. In that manner, for each candidate, the system associated the most similar compound that causes the signaling with its respective target information, such as potency, types of assay, organism, etc. This information can be useful in evaluating the importance of the candidate and also facilitates experimental validation. In our study, we focused on the evaluation of natural products and experimental or existing drugs. However, the use of additional synthetic compounds in the initial data was very useful to provide a frame of reference to calculate similarity and implement new candidate targets for drugs that would not be available using databases with only existing drugs.

We have shown that our TIPF model is very useful for large scale screening. In Figure S1 we showed that our initial model retrieved an enriched set of COX-1 candidates from the point of view of the docking score. In a second step and as a refinement procedure, some candidates can be studied through methodologies such as molecular docking to make a final candidate selection for experimental validation. Alternative similarity measures between pairs of molecules could be used in the development of methods of computational screening, such as structural similarity, adverse effect profiles or gene expression similarities, among others^14^,^22^. However, target profiles comparison is simple and efficient and big data sources are available for the scientific community, such as ChEMBL^25^ or PubChem^40^.

Pairs of compounds identified by our model with good target similarity scores could share many structural patterns or belong to the same structural family. However, our system was also able to identify pairs of compounds not similar from the point of view of the molecular structure similarity, but still similar according to the protein interaction profiles. Figure S2 shows 2D molecular similarity for a random set of 500 pairs of molecules in our model with a TC ≥ 0.85 using TIPFs. Moreover, ranges of similarity are dependent on the measure taken into account. Two molecules with a TC = 0.5 using structural molecular fingerprints, such as MACCS, could be considered not similar. Two molecules with TC = 0.5 using TIPFs could be deemed as similar since they share around half of the protein interactions in common, with the ability of following similar biological pathways.

Our large scale multi-target predictor showed potential in lead discovery and repurposing. Moreover, applicability in drug safety could be exploited. As an example, lapatinib showed moderate hCOX-1 inhibitory activity. Interaction with this enzyme is related with a higher risk of bleeding. No reports of bleeding were found with the use of the drug lapatinib but other tyrosine kinase inhibitors such as dasatinib, sunitinib, sorafenib and ponatinib, are associated with higher risk of bleeding^41^,^42^,^43^. Although interaction with hCOX-1 could be a possible mechanism of action that contributes to the adverse effect, experimental studies are necessary to provide evidences to this hypothesis.

## Methodology

### Target interaction profiles modeling

#### Ligands/targets database

The set of ligands also including drugs in the market, experimental drugs and natural compounds, was extracted from ChEMBL database, a multiple source of compounds, targets and bioassays data^25^. The data was pre-processed as a previous step of modeling. This step included elimination of no single or unspecified proteins, repeated cases, not specific biological data, such as cases reporting “not active”, “not determined”, “not soluble”, “potential missing data”, etc. Not high affinity compound-target cases, such as cases when the potency of the compounds was established as greater than 50,000 nM, were excluded. Different bioassays including the same protein were clustered in the same case target. Additional information for each case, such as types of bioassay, organism, etc, was also collected. Final compound-target data was transformed in a matrix representing compounds (rows) and targets or proteins (columns). Each cell presented a value of 1 if the compound-target is included in our database and a value of 0 when the case is not incorporated to the data. With the aim of generating robust data, we considered only compounds with at least 15 targets. The final data set comprised 449,996 drug-target positive cases out of a total of 28,846,904 possible drug-target combinations (11,548 compounds and 2,498 different targets).

#### Target interaction profile fingerprints (TIPFs)

From the matrix representing our compound-target data, we calculated a TIPF for each compound (data only calculated for compounds with at least 15 targets). The concept of TIPFs is analogous to the molecular fingerprints that codify in each vector position a structural pattern^14^,^44^. In our TIPFs, each bit position of the vector codified the interaction (value 1) or non-interaction (value 0) with the different targets (see Fig. 2 for a graphical description). As an efficient manner of representing a sparse binary bit vector, only the positions representing interactions were stored in the final fingerprints.

#### Computation of fingerprint similarity

We calculated the Tanimoto coefficient (TC) to compute similarity between all the TIPF pairs. The TC is defined in our case as the ratio between the protein interactions in the intersection and the union of the pair of fingerprints (see Fig. 2):


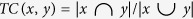


#### Compound-target modeling

Our predictor exchanged the protein interactions between each pair of compounds and associated the TC to the compound-target associations. The same compound-target case can be generated from the comparison of different pairs. However, only the maximum TC score was retained for the candidate. It means that the similarity of each compound-target case was evaluated against all the compounds that are described to interact with the target in ChEMBL. Retrieval of known cases was measured to assess performance in the model. Sensitivity, specificity, precision and EF were measured at different TC thresholds as quality parameters. Performance was also evaluated through hold-out validation series.

### Molecular docking simulations

Molecular docking simulations were carried out in the hMAO-B and COX-1 (organism *Ovis aries*) enzymes with Glide from the Schrödinger package^30^.

#### hMAO-B docking

Different sets of ligands were included in the docking: the set of 13 drugs and natural compounds selected though TIPF modeling to further study, a set of 9 co-crystallized compounds extracted from the Protein Data Bank^45^, and a set of 1,126 drug decoys extracted from DrugBank^46^. The set of decoys was selected according to the following rules to assure certain similarity with the co-crystallized molecules and set of candidates: drugs with 15–60 atoms, topological polar surface area not greater than 200, and not more than one violation to the drug-like Lipinski´s rules. All the ligands were prepared with the LigPrep module. This step included the generation of tautomers and different protonation states at pH = 7.0 ± 2.0, and initial optimization of the molecular geometry.

Two crystallized hMAO-B proteins, downloaded from the PDB (PDB codes: 3PO7^29^ and 2V60^31^), were selected to run the docking simulations. 3PO7 contains the hMAO-B protein co-crystallized with the drug zonisamide. In 2V60, the protein is co-crystallized with the inhibitor 7-(3-chlorobenzyloxy)-4-carboxaldehydecoumarin (c17). Different calculations without and with water molecules in the pocket were performed: 5 water molecules in the protein pocket (HOH545, HOH566, HOH581, HOH590, HOH846 in 3PO7; HOH1159, HOH1166, HOH1171, HOH1206, HOH1309 in 2V60), including waters in the pocket establishing hydrogen bonds with the co-crystallized ligands (1 water molecule in 2V60 and 2 waters in 3PO7), including water molecules in a distance of 5 Å from the ligands, and excluding water molecules in the protein pocket. Protein structures were pre-processed with the Protein Preparation Workflow^30^ that added hydrogens and cap termini, optimized protonation states of the residues and optimized the hydrogen network.

In a first step, a receptor grid was calculated in each protein and centered in the co-crystallized ligands (van der Waals scaling factor = 1, partial charge cut-off = 0.25). Then, docking of the compounds into the different hMAO-B pockets were carried out with Glide SP (standard precision) from Schrödinger. Five poses for each ligand were retained. Final pose selection was carried out with Emodel. The docking was validated measuring the root mean square deviation (RMSD) of the heavy atoms coordinates between theoretical poses extracted from the calculation and co-crystallized conformations of 9 ligands (PDB: 3PO7, 1OJ9, 1OJA, 2BK3, 2V5Z, 2V60, 2V61, 2XFN, 4A79)^45^ (see Table S3 of the Supporting Information with RMSD values). Additionally, we evaluated the ability of the system to prioritize ligands (9 co-crystallized compounds) over non-ligands (set of drug decoys extracted from DrugBank) using ROC curves (AUROC values in Table S4 of the Supporting Information).

#### COX-1 docking

A similar protocol and parameters as described above were used for the COX-1 docking. The set of ligands (including 384 candidates and 8 co-crystallized compounds) were prepared with LigPrep. We downloaded the protein crystal structure 3KK6 (PDB code)^34^. The protein belongs to the organism *Ovis aries*, since not human protein was available. Water molecules were removed and protein structure was pre-processed with the Protein Preparation Workflow. The receptor grid was centered in the co-crystallized inhibitor celecoxib. We run molecular docking simulations with Glide SP (standard precision) from Schrödinger^30^. Final pose selection was carried out with the energetic parameter Emodel. The docking was validated measuring the RMSD between co-crystallized conformations and theoretical poses for 8 inhibitors (see Table S5 of the Supporting Information with RMSD values). The area under the ROC curve was 0.7 showing the capacity to prioritize true ligands (8 co-crystallized compounds) over non-ligands (set of drug decoys extracted from DrugBank).

### Determination of enzymatic activity towards MAO isoforms

The biological evaluation on hMAO activity was investigated by measuring effects on the production of hydrogen peroxide (H_2_O_2_) from *p*-tyramine, using the Amplex^®^ Red MAO assay kit and microsomal isoforms prepared from insect cells (BTI-TN-5B1-4) infected with recombinant baculovirus containing cDNA inserts for hMAO-A and hMAO-B. The production of H_2_O_2_ catalysed by the MAO isoforms was detected using 10-acetyl-3,7-dihydroxyphenoxazine (Amplex^®^ Red reagent), a non fluorescent and highly sensitive probe that reacts with H_2_O_2_ in the presence of horseradish peroxidase to produce a fluorescent product, resorufin. The selected compounds and reference inhibitor were unable to react directly with the Amplex^®^ Red reagent, which indicates that these drugs do not interfere with the measurements. Under the experimental conditions hMAO-A displayed a Michaelis constant (K_m_) equal to 457.17 μM and maximum reaction velocity (V_max_) in the control group of 185.67 ± 12.06 (nmol p-tyramine/min)/mg protein whereas hMAO-B showed a K_m_ of 220.33 ± 32.80 μM and V_max_ of 24.32 ± 1.97 (nmol*p*-tyramine/min)/mg protein (n = 5). Most tested compounds concentration-dependently inhibited this enzymatic control activity.

The selected compounds were dissolved in DMSO (Sigma-Aldrich, Alcobendas, Madrid, Spain) to prepare 10 mM stock solutions, which were kept for storage at −20 °C. Percentage of DMSO used in the experiments was never higher than 1%. Human recombinant MAO isoforms (native enzymes), used in the experiments, were purchased from Sigma-Aldrich (Alcobendas, Madrid, Spain). Test compounds were acquired from Sigma-Aldrich and Vitro S.A. (Madrid, Spain).

Briefly, 0.1 mL of sodium phosphate buffer (0.05 M, pH 7.4) containing different concentrations of the test drugs (selected compounds or reference inhibitor) and adequate amounts of recombinant hMAO-A or hMAO-B required and adjusted to obtain in our experimental conditions the same reaction velocity, that is, to oxidize (in the control group) the same concentration of substrate: 165 pmol of p-tyramine/min (hMAO-A: 1.1 μg protein; specific activity: 150 nmol of p-tyramine oxidized to p-hydroxyphenylacetaldehyde/min/mg protein; hMAO-B: 7.5 μg protein; specific activity: 22 nmol of *p*-tyramine transformed/min/mg protein) were incubated for 15 min at 37 °C in a flat-black-bottom 96-well microtest plate, placed in the dark fluorimeter chamber. After this incubation period, the reaction was started by adding (final concentrations) 200 μM Amplex^®^ Red reagent, 1 U/mL horseradish peroxidase and 1 mM*p*-tyramine. The production of H_2_O_2_ and, consequently, of resorufin was quantified at 37 °C in a multi-detection microplate fluorescence reader (FLX800, Bio-Tek Instruments, Inc., Winooski, VT, USA) based on the fluorescence generated (excitation, 545 nm, emission, 590 nm) over a 15 min period, in which the fluorescence increased linearly. Control experiments were carried out simultaneously by replacing the test drugs (new compounds and reference inhibitor) with appropriate dilutions of the vehicles. In addition, the possible capacity of the above test drugs to modify the fluorescence generated in the reaction mixture due to non-enzymatic inhibition (e.g., for directly reacting with Amplex^®^ Red reagent) was determined by adding these drugs to solutions containing only the Amplex^®^ Red reagent in a sodium phosphate buffer. To determine the kinetic parameters of hMAO (K_m_ and V_max_), the corresponding enzymatic activity was evaluated (under the experimental conditions described above) in the presence of a number (a wide range) of *p*-tyramine concentrations.

The specific fluorescence emission (used to obtain the final results) was calculated after subtraction of the background activity, which was determined from wells containing all components except the hMAO, which were replaced by a sodium phosphate buffer solution. In our experimental conditions, this background activity was practically negligible.

MAO activity of the test compounds and reference inhibitor is expressed as IC_50_, e.g. the concentration of each drug required to produce a 50% decreased on control value activity isoform MAO.

### Determination of enzymatic activity towards hCOX-1

#### Preparation of microsomes from human platelets

Human platelets were isolated by centrifugation from buffy coats obtained from the Centro de Transfusion de Galicia (Santiago de Compostela, Spain) and prepared as described. Briefly, the buffy coat was diluted 1:1 with washing buffer of the following composition at pH 6 (mM): NaCl (120), KCl (5), trisodium citrate (12), glucose (10), sucrose (12.5), and then centrifuged at 400 g for 8 min in a centrifuge (Omnifuge 2.0 RS, Heraeus Sepatech, Osterade, Germany) at 25 °C to obtain platelet rich plasma.

The upper layer obtained in this centrifugation, containing platelet rich plasma, was gently removed and centrifuged at 850 g for 20 min at 4 °C in a centrifuge (J2-MI, Beckman Instruments, Inc., Palo Alto, California, USA). The platelet pellet was recovered, resuspended with washing buffer, and centrifuged again at 850 g for 20 min at 4 °C. To prepare human platelet microsomes, the resultant platelet pellet of the above centrifugation was resuspended in 7 mL of sodium phosphate buffer (10 mM, pH 7.4), sonicated at 50 W for 50 s (5 pulses of 10 s), and centrifuged at 850 g for 20 min at 4 °C in a refrigerated centrifuge. The pellet was discarded and the supernatant was subsequently centrifuged at 10 000 g for 10 min at 4 °C in the same centrifuge. The pellet obtained in this centrifugation was discarded and the supernatant was finally centrifuged at 100 000 g for 1 h at 4 °C in an ultracentrifuge (Beckman Instruments, Inc., Palo Alto, California, USA).

The resultant pellet containing platelet microsomes was resuspended in 1 mL of sodium phosphate buffer (50 mM, pH 7.4) and the protein concentration in the platelet microsome suspension (approximately 2 mg/mL) was measured by the method of Bradford, using a protein assay kit from BioRad Laboratories (Alcobendas, Spain). Platelet microsome aliquots were stored at 80 °C for several days (without apparent loss of COX activity) until use.

#### Determination of hCOX-1 activity

The biological evaluation of the test drugs on hCOX-1 activity (bisdioxygenase and peroxidase reactions) was investigated by measuring their effects on the oxidation of N, N, N’, N’-tetramethyl-*p*-phenylenediamine (TMPD) to N, N, N’, N’-tetramethyl-*p*-phenylene-diimine, using arachidonic acid as substrate for hCOX-1 from human platelet microsomes (obtained as described in the above paragraph). The formation of N, N, N’, N’-tetramethyl-p-phenylenediimine (a coloured compound) from N, N, N’, N’-tetramethyl-p-phenylenediamine catalyzed by COX can be detected spectrophotometrically at 600 nm. In this study, hCOX activity was evaluated using a spectrophotometric method. Briefly, 0.1 mL of Tris–HCl buffer (100 mM, pH 8) containing 1 mM hematin, 100 mM TMPD, various concentrations of the test drugs, and appropriate amounts of hCOX-1 (0.08 A600 U/min) were incubated for short periods of time (3–5 min) to avoid a notable loss of COX activity at 37 °C in a flat-bottom 96-well microtest plate (BD Biosciences, Franklin Lakes, NJ, USA) placed in the dark multimode microplate reader chamber. After this incubation period, the reaction was started by adding (final concentration) 100 mM arachidonic acid and the formation of N, N,N’,N’-tetramethyl-p-phenylenediimine from TMPD, i.e., the increase in absorbance at 600 nm was measured at 37 °C in a multi-mode microplate reader (Fluostar Optima, BMG Labtech GmbH, Offenburg, Germany) for 25 s, aperiod in which the absorbance increased linearly from the beginning. The specific absorbance (used to obtain the final results) was calculated after subtraction of the background absorbance generated in wells containing a blank solution, i.e., all components except the COX isoform, which were replaced by a Tris–HCl buffer solution. Under our experimental conditions, this background activity was practically negligible.

Control experiments were carried out simultaneously by replacing the test drugs (new compounds and reference inhibitors) with appropriate dilutions of the vehicles. In addition, the possible capacity of the above test drugs to modify the absorbance of the reaction mixture due to non-enzymatic inhibition (e.g., for directly reacting with TMPD) was determined by adding these drugs to solutions containing only TMPD in a Tris–HCl buffer solution.

## Additional Information

**How to cite this article**: Vilar, S. *et al.* Computational Drug Target Screening through Protein Interaction Profiles. *Sci. Rep.* **6**, 36969; doi: 10.1038/srep36969 (2016).

**Publisher’s note**: Springer Nature remains neutral with regard to jurisdictional claims in published maps and institutional affiliations.

## Supplementary Material

Supplementary Information

## Figures and Tables

**Figure 1:**
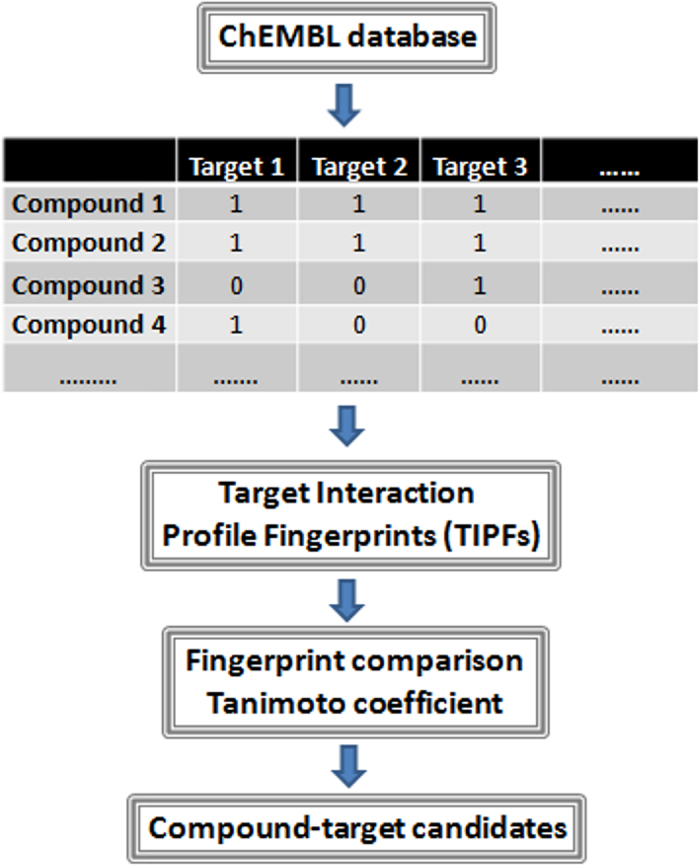
Flowchart of the main steps involved in the development of the compound-target predictor

**Table 1:** Drug/natural compound candidates extracted from the TIPF virtual screening and molecular docking.

HMAO-B CANDIDATE	DOCKING RANKING	REFERENCE COMPOUND CHEMBL	TC
PIPERLONGUMINE	1	CHEMBL339561	0.50
FRENTIZOLE	2	CHEMBL339561	0.50
ETHOXZOLAMIDE	3	CHEMBL750 (ZONISAMIDE)	0.58
**HCOX-1 CANDIDATE**	**DOCKING RANKING**	**REFERENCE COMPOUND CHEMBL**	**TC**
LAPATINIB	9	CHEMBL10	0.88
SB-202190	16	CHEMBL10	0.83
RO-316233	30	CHEMBL35482	0.84
GW786460X	46	CHEMBL35482	0.54
INDIRUBIN-3′-MONOXIME	74	CHEMBL35482	0.91

Candidates are predicted to interact with hMAO-B and hCOX-1. Docking ranking is also shown along with the most similar compound in the reference standard (ChEMBL) and the Tanimoto coefficient (TC).

**Table 2:** *IC*_50_ values for the inhibitory effects of the test drugs (compounds and reference inhibitors) on the enzymatic activity of human MAO-B (rational assays selected by our modeling), human MAO-A (control assays not selected by the modeling) and human COX-1 (control assays not selected by the modeling).

DRUG	*IC*_*50*_ hMAO-B (*μ*M)	*IC*_*50*_ hMAO-A (*μ*M)	*IC*_*50*_ hCOX-1 (*μ*M)
Piperlongumine	65.07 ± 2.99	**	**
Frentizole	**	**	**
Ethoxzolamide	24.57 ± 1.09	**	**
Selegiline (Reference inhibitor)	0.019 ± 0.001		
Iproniazide (Reference inhibitor)	7.54 ± 0.36	6.56 ± 0.76	
Moclobemide (Reference inhibitor)		0.36 ± 0.02	
FR122047 (Reference inhibitor)			0.094 ± 0.006

Each *IC*_50_ value is the mean ± S.E.M. from three experiments (n = 3). ** Inactive at 100 μM (highest concentration tested).

**Table 3:** *IC*_50_ values for the inhibitory effects of the test drugs (compounds and reference inhibitors) on the enzymatic activity of human COX-1 (rational assays selected by our modeling) and human MAO-B (control assays not selected by the modeling).

DRUG	*IC*_*50*_hCOX-1 (*μ*M)	*IC*_*50*_ hMAO-B (*μ*M)
Lapatinib	^a^19.5 % inhibition at 25 μM	**
SB-202190	23.59 ± 3.53	**
RO-316233	25.35 ± 3.80	**
GW786460X	**	**
Indirubin-3′-monoxime	^a^28 % inhibition at 25 μM	**
Iproniazide (Reference inhibitor)		7.54 ± 0.36
Selegiline (Reference inhibitor)		0.019 ± 0.001
FR122047 (Reference inhibitor)	0.094 ± 0.006	
Indomethacin (Reference inhibitor)	12.16 ± 1.16	
Diclofenac (Reference inhibitor)	18.23 ± 1.73	

Each *IC*_50_ value is the mean ± S.E.M. from three experiments (n = 3). ** Inactive at 100 μM (highest concentration tested). ^a^ % enzymatic inhibition at 25 μM (compounds precipitated at higher concentrations).
