# Supplementary material for: Computational Drug Target Screening through Protein Interaction Profiles
Source: Sci Rep. 2016 Nov 15;6:36969. doi: 10.1038/srep36969 (PMC5109486; doi:10.1038/srep36969)

**Computational Drug Target Screening through Protein Interaction Profiles**

Santiago Vilar^1,2*^, Elías Quezada^3^, Eugenio Uriarte^2^, Stefano Costanzi^4^, Fernanda Borges^3^, Dolores Viña^5^ and George Hripcsak^1*^

^1^ Department of Biomedical Informatics, Columbia University Medical Center, New York, NY 10032, USA

^2^Department of Organic Chemistry, Faculty of Pharmacy, University of Santiago de Compostela, 15782 Santiago de Compostela, Spain

^3^CIQUP, Department of Chemistry & Biochemistry, Faculty of Sciences, University of Porto, 4169-007 Porto, Portugal

^4^Department of Chemistry, American University, 20016 Washington, DC, USA

^5^Department of Pharmacology, CIMUS, University of Santiago de Compostela, 15782 Santiago de Compostela, Spain

**Supporting Information**

**Table S1.** SP scoring function (standard-precision, kcal/mol) extracted from molecular docking simulations for the 13 initial drug candidates in the hMAO-B. It is shown in red color the first three scored candidates in each calculation. In some cases the docking calculation did not yield any pose for the candidate (N/A in the table).

| **CANDIDATES** | **3PO7**  **(5 H2O molecules)^a^** | **3PO7**  **(2 H2O)^b^** | **3PO7**  **(5Å H2O)^c^** | **3PO7**  **(no H2O)^d^** | **2V60**  **(5 H2O molecules)^e^** | **2V60**  **(1 H2O)^f^** | **2V60**  **(5Å H2O)^g^** | **2V60**  **(no H2O)^h^** |
| --- | --- | --- | --- | --- | --- | --- | --- | --- |
| **PIPERLONGUMINE** | -9.94 | -8.86 | -9.57 | -9.22 | -9.28 | -8.99 | -9.34 | -8.90 |
| **FRENTIZOLE** | -9.02 | -7.92 | -8.82 | -9.39 | -8.88 | -8.67 | -8.48 | -8.72 |
| **ETHOXZOLAMIDE** | -8.19 | -7.27 | -8.17 | -8.26 | -7.87 | -7.80 | -7.15 | -8.05 |
| **MAFENIDE** | -7.64 | -7.59 | -7.24 | -7.92 | -7.64 | -8.10 | -8.42 | -7.56 |
| **METHAZOLAMIDE** | -7.52 | -6.57 | -7.07 | -6.64 | -7.47 | -6.45 | -5.94 | -6.54 |
| **SULFANILAMIDE** | -7.28 | -7.00 | -7.13 | -7.10 | -7.13 | -6.93 | -6.67 | -6.86 |
| **BRINZOLAMIDE** | -7.15 | -7.15 | -8.24 | -7.33 | -7.24 | -7.61 | N/A | -7.15 |
| **HISTIDINE** | -5.76 | -5.96 | -6.24 | -5.57 | -6.02 | -5.50 | -5.70 | -5.85 |
| **DORZOLAMIDE** | -5.41 | -5.20 | -7.23 | -7.82 | -8.00 | -5.73 | N/A | -5.57 |
| **CUCURBITACIN B** | N/A | N/A | N/A | N/A | N/A | N/A | N/A | N/A |
| **STREPTONIGRIN** | N/A | N/A | N/A | N/A | N/A | N/A | N/A | N/A |
| **TOPIRAMATE** | N/A | N/A | N/A | N/A | N/A | N/A | N/A | N/A |
| **VINBLASTINE** | N/A | N/A | N/A | N/A | N/A | N/A | N/A | N/A |

^a^ 3PO7 crystal structure with 5 water molecules in the pocket (HOH545, HOH566, HOH581, HOH590, HOH846).

^b^ 3PO7 crystal structure with 2 water molecules establishing H-bonds with the co-crystallized ligand in the pocket.

^c^ 3PO7 crystal structure with water molecules in a distance of 5Å from the co-crystallized ligand.

^d^ 3PO7 crystal structure with no water molecules in the pocket.

^e^ 2V60 crystal structure with 5 water molecules in the pocket (HOH1159, HOH1166, HOH1171, HOH1206, HOH1309).

^f^ 2V60 crystal structure with 1 water molecule establishing H-bond with the co-crystallized ligand in the pocket.

^g^ 2V60 crystal structure with water molecules in a distance of 5Å from the co-crystallized ligand.

^h^ 2V60 crystal structure with no water molecules in the pocket.

**Table S2.** SP scoring function (standard-precision) extracted from molecular docking simulations for the 5 drug candidates selected for experimental validation in COX-1. Docking ranking among the set of 384 candidates is also shown. Docking ranking and commercial availability were considered in the final selection. The most similar compound in our ChEMBL reference standard calculated with our TIPF model is provided.

| **CANDIDATES** | **SP (kcal/mol)** | **Docking ranking** | **Reference compound ChEMBL** |
| --- | --- | --- | --- |
| **LAPATINIB** | -10.44 | 9 | CHEMBL10 |
| **SB-202190** | -10.01 | 16 | CHEMBL10 |
| **RO-316233** | -9.69 | 30 | CHEMBL35482 |
| **GW786460X** | -9.46 | 46 | CHEMBL35482 |
| **INDIRUBIN-3’-MONOXIME** | -9.05 | 74 | CHEMBL35482 |

**Table S3.** Root mean square deviation (RMSD) of the heavy atoms coordinates between co-crystallized ligands and theoretical poses extracted from Glide SP docking calculations in the hMAO-B. RMSD>2.5 Å is highlighted in red (poor crystallographic conformation retrieval).

| **RMSD (Å)** | | | | | | | | |
| --- | --- | --- | --- | --- | --- | --- | --- | --- |
| **PDB code** | **3PO7**  **(5 H2O molecules)^a^** | **3PO7**  **(2 H2O)^b^** | **3PO7**  **(5Å H2O)^c^** | **3PO7**  **(no H2O)^d^** | **2V60**  **(5 H2O molecules)^e^** | **2V60**  **(1 H2O)^f^** | **2V60**  **(5Å H2O)^g^** | **2V60**  **(no H2O)^h^** |
| **3PO7** | 0.27 | 1.06 | 1.05 | 2.68 | 0.34 | 2.66 | 3.10 | 2.66 |
| **1OJA** | 0.38 | 8.88 | 2.95 | 8.90 | 9.20 | 9.20 | 4.77 | 9.20 |
| **1OJ9** | 0.59 | 0.62 | 1.54 | 0.76 | 0.79 | 0.76 | 1.82 | 1.74 |
| **2V5Z** | 0.80 | 1.67 | 1.51 | 0.73 | 0.81 | 0.92 | 9.03 | 0.60 |
| **2V60** | 1.03 | 0.26 | 0.45 | 1.02 | 0.52 | 0.90 | 0.37 | 0.89 |
| **2BK3** | 1.21 | 1.73 | 1.97 | 8.61 | 1.55 | 1.76 | 2.10 | 8.34 |
| **2V61** | 1.47 | 8.41 | 8.40 | 1.42 | 1.40 | 1.43 | 3.21 | 1.39 |
| **4A79** | 3.20 | 9.64 | 3.14 | 3.64 | 3.11 | 3.37 | 9.38 | 10.13 |
| **2XFN** | 6.98 | 6.74 | 6.40 | 8.72 | 1.71 | 1.71 | 6.37 | 4.25 |

^a^ 3PO7 crystal structure with 5 water molecules in the pocket (HOH545, HOH566, HOH581, HOH590, HOH846).

^b^ 3PO7 crystal structure with 2 water molecules establishing H-bonds with the co-crystallized ligand in the pocket.

^c^ 3PO7 crystal structure with water molecules in a distance of 5Å from the co-crystallized ligand.

^d^ 3PO7 crystal structure with no water molecules in the pocket.

^e^ 2V60 crystal structure with 5 water molecules in the pocket (HOH1159, HOH1166, HOH1171, HOH1206, HOH1309).

^f^ 2V60 crystal structure with 1 water molecule establishing H-bond with the co-crystallized ligand in the pocket.

^g^ 2V60 crystal structure with water molecules in a distance of 5Å from the co-crystallized ligand.

^h^ 2V60 crystal structure with no water molecules in the pocket.

**Table S4.** AUROC results for the different molecular docking simulations in the hMAO-B using the 2V60 and 3PO7 crystal structures (hMAO-B). True positives fraction is represented by the 9 crystallized ligands (see Table 1) and the false positive fraction is represented by the set of drug decoys extracted from DrugBank. Calculations were performed with SP (standard precision) of Glide. SP (5 water_molecules) included 5 water molecules in the protein pocket (HOH1159, HOH1166, HOH1171, HOH1206, HOH1309 in 2V60; HOH545, HOH566, HOH581, HOH590, HOH846 in 3PO7). SP (ligand H-bond waters) included waters in the pocket establishing hydrogen bonds with the co-crystallized ligand (1 water molecule in 2V60 and 2 waters in 3PO7). SP (5Å waters) included water molecules in a distance of 5Å from the ligands. SP (no water) did not include water molecules in the protein pocket.

| **AUROC** | | |
| --- | --- | --- |
|  | **PDB: 3PO7** | **PDB: 2V60** |
| **SP (5 water_molecules)** | 0.76 | 0.77 |
| **SP (ligand H-bond waters)** | 0.75 | 0.75 |
| **SP (5Å waters)** | 0.75 | 0.69 |
| **SP (no water)** | 0.74 | 0.74 |

**Table S5.** Root mean square deviation (RMSD) of the heavy atoms coordinates between co-crystallized ligands and theoretical poses extracted from Glide SP docking calculations in the COX-1. RMSD>2.5 Å is highlighted in red (poor crystallographic conformation retrieval).

| **COX-1** | |
| --- | --- |
| **PDB code** | **RMSD**  **(Å)** |
| **1EQG** | 1.81 |
| **1HT8** | 0.61 |
| **1PGE** | 2.06 |
| **3KK6** | 0.85 |
| **3N8X** | 1.01 |
| **3N8Y** | 5.87 |
| **3N8Z** | 0.75 |
| **4O1Z** | 3.83 |

**Figure S1.** Histogram of docking scores for the set of COX-1 candidates extracted from our TIPF model compared with a set of drugs extracted from DrugBank (number of observations versus docking score; *p*-value<0.05, Kolmogorov-Smirnov comparison of two data sets).


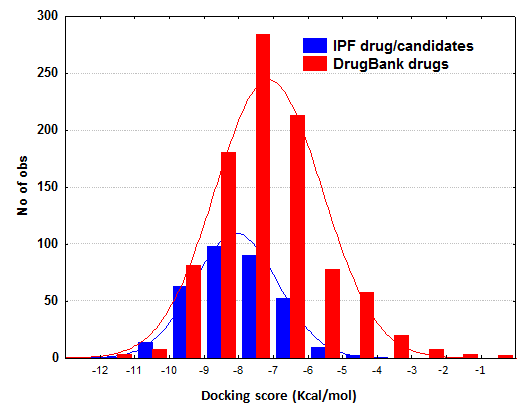


**Figure S2.** 2D molecular similarity for a random set of 500 pairs of molecules in our model with a TC≥0.85 using TIPFs. TC-MACCS is the Tanimoto coefficient calculated for each pair of 2D MACCS fingerprints. TC-TIPF is the Tanimoto coefficient calculated for each pair of Target Interaction Profile Fingerprints.


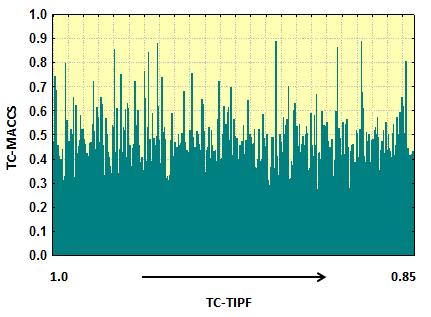

Supplement: Supplementary Information [file srep36969-s1.doc]
